# The *Klebsiella pneumoniae* carbapenemase (KPC) β-Lactamase Has Evolved in Response to Ceftazidime Avibactam

**DOI:** 10.3390/antibiotics13010040

**Published:** 2023-12-31

**Authors:** Sona Garsevanyan, Miriam Barlow

**Affiliations:** Department of Molecular and Cell Biology, School of Natural Sciences, University of California Merced, Merced, CA 95343, USA; sgarsevanyan@ucmerced.edu

**Keywords:** ceftazidime, avibactam

## Abstract

*Klebsiella pneumoniae* carbapenemase KPC is an important resistance gene that has disseminated globally in response to carbapenem use. It is now being implicated as a resistance determinant in Ceftazidime Avibactam (CAZ-AVI) resistance. Given that CAZ-AVI is a last-resort antibiotic, it is critical to understand how resistance to this drug is evolving. In particular, we were interested in determining the evolutionary response of KPC to CAZ-AVI consumption. Through phylogenetic reconstruction, we identified the variable sites under positive selection in the KPC gene that are correlated with Ceftazidime Avibactam (CAZ-AVI) resistance. Our approach was to use a phylogeny to identify multiple independent occurrences of mutations at variable sites and a literature review to correlate CAZ-AVI resistance with the mutations we identified. We found the following sites that are under positive selection: P104, W105, A120, R164, L169, A172, D179, V240, Y241, T243, Y264, and H274. The sites that correlate with CAZ-AVI resistance are R164, L169, A172, D179, V240, Y241, T243, and H274. Overall, we found that there is evidence of positive selection in KPC and that CAZ-AVI is the major selective pressure.

## 1. Introduction

Resistance to carbapenems among Enterobacteriaceae was first reported in the early 1990s, and since then it has increased greatly. It has become a worldwide problem and a serious challenge to global health [1,2]. Carbapenem resistance is mainly caused by three classes of β-lactamases, as defined by Ambler: Class A is a group of serine β-lactamases that mainly confer resistance to penicillins and cephalosporins. However, a few members of this group confer carbpenem resistance, including *Klebsiella pneumoniae* carbapenemase (KPC) and *Guiana Extended* Spectrum (GES) β-lactamase. Class B does not share homology with the other β-lactamase groups and has a different active site that contains a zinc ion. For this reason, they are called metallo-β-lactamases. While this group exhibits some activity against other β-lactam antibiotics, its clinical importance derives almost entirely from its activity against carbapenems. This group includes the members Imipenemase (IMP), Verona Integron-borne metallo-β-lactamase (VIM), and the New Delhi metallo-β-lactamase (NDM) that frequently result in carbapenem treatment failure. Class D serine β-lactamases are the third group. This group comprises oxacillinase hydrolyzing enzymes (OXA) and features some, such as *oxacillinase*-48 (OXA-48), that also exhibit carbamenemase activity [3,4].

Among Class A carbapenemases, there are a variety located on the chromosome that encode the enzymes NmcA, SME, IMI-1, SFC-1, BIC-1, PenA, FPH-1, and SHV-38. There are also Class A carbapenemases located on plasmids encoding the enzymes KPC, IMI-2, GES, and FRI-1. CRE infections in health-care settings and acute care hospitals are an ongoing challenge when *K. pneumoniae* expresses a carbapenemase. One common carbapenemase gene found in *K. pneumoniae* is the *Klebsiella pneumoniae* carbapenemase (*bla*_KPC_) [5].

Among CREs in the US and China, the most common determinants of carbapenem resistance are *Klebsiella pneumoniae* carbapenemases (KPC), especially *bla*_KPC-2_ and *bla*_KPC-3_ [6,7]. These are plasmid-mediated Class A carbapenemases. KPC has evolved and spread rapidly since *bla*_KPC-2_ was first identified in 1996 [8], likely due to high carbapenem consumption. In addition to its dissemination on plasmids, it has also evolved a wide range of variants with unique amino acid sequences [9,10,11,12]. Its resistance capabilities include cephalosporin hydrolysis, inhibitor resistance, and carbapenem hydrolysis [13]. As the *bla*_KPC_ gene has disseminated across the earth in response to carbapenem consumption, the stage has been set for CAZ-AVI resistance.

KPC was first identified in a *Klebsiella pneumoniae* isolate in 1996 from North Carolina [8]. Within a few years, KPC-2 and KPC-3 started causing outbreaks in multiple hospitals across New York City [12]. A surveillance study from 2002 to 2003 in New York City found 9 out of 602 *K. pneumoniae* isolates contained a *bla*_KPC_ gene. KPC has also been found in *E. coli*. KPC has also been identified in many members of the Enterobacteriaceae family, such as *Escherichia coli*, *Klebsiella oxytoca*, *Salmonella enterica*, *Citrobacter freundii*, *Enterobacter* spp., and *Serratia marcescens* [14]. These enzymes were mainly found in Enterobacteriaceae until 2007, when a few *Pseudomonas aeruginosa* isolates expressing KPC were found in Medellin, Colombia [14]. These isolates were highly resistant to carbapenems. Similar isolates can now be found in America, South America, Asia, and Europe [15].

The *bla*_KPC-2_ gene was identified on a Tn3-based transposon, Tn4401, which is 10 kb in size, and it is known to be a very active transposon on many different plasmids [15,16]. Tn4401 is marked with two inverted repeat sequences, carries transposase and resolvase genes, along with two insertion sequences, ISK*pn*6 and ISK*pn*7, and additionally *bla*_KPC_ [16]. Previously published work shows that in different genetic environments, there are other sequences upstream of *bla*_KPC_, but the downstream sequences are similar to Tn4401 [16]. Additionally, *bla*_KPC_ has been found on a variety of plasmids varying in size, length, transposon location, self-transferable plasmids, number of copies, and efficient STs [17]. This has contributed to the mobilization, rapid dissemination, and spread of KPC-associated carbapenem resistance [2,17].

*bla*_KPC_ has also been found on Tn4401 negative plasmids and on different transposons called NTEKPC (non-Tn4401 elements) [18]. Jousset et al. also noted pKpQIL-like plasmids to be very compatible with the *K. pneumoniae* clonal group 258 (CG258) as another contributing factor to the worldwide dissemination of *bla*_KPC_. The dissemination of *bla*_KPC_ is mainly associated with the clonal group 258 (CG258) and localization in different plasmids and transposons, particularly in the Tn4401 transposon variants [15,17,19,20,21].

The abundance of carbapenem-resistant clinical isolates necessitated the development of treatments that would be effective against carbapenem-resistant Gram-negative bacteria. In 2015, a non-β-lactam β-lactamase inhibitor called avibactam (AVI) was released to be given as treatment in combination with ceftazidime (CAZ), a third-generation cephalosporin that was approved for clinical treatment in the USA [22]. It was initially an effective treatment for carbapenemase-producing Enterobacterales, making it the first-line antibiotic treatment for multidrug-resistant bacteria [23,24]. Since ceftazidime avibactam (CAZ-AVI) was approved for clinical use, resistance to the drug has increased drastically [23]. Gram-negative bacteria exhibiting carbapenem resistance are no longer uniformly susceptible to CAZ-AVI. This is largely due to the fact that KPC has emerged as a major player in CAZ-AVI resistance. In China, CAZ-AVI was approved for clinical use in 2019, and since then, CAZ-AVI resistance has increased significantly [22].

CREs are resistant to CAZ-AVI, especially when they express *bla*_KPC_. Mutations that increase CAZ-AVI resistance can be observed in the Ω-loop of KPC-2 and KPC-3 [22]. However, there are other genes likely contributing to resistance. CAZ-AVI resistance in *E. coli* has been reported in very low quantities [25]. When *E. coli* is resistant to CAZ-AVI, it is often due to a mutation in one of the following: *bla*_KPC_, *bla*_SHV_, *bla*_CTX-M_, and *bla*_OXA_ resistance genes [25,26,27,28,29,30].

There is also precedent for the involvement of non-carbapenemase β-lactamases in CAZ-AVI resistance. A study by Shields et al. found plasmid-borne *bla*_KPC-3_ mutations appeared while three patients were in treatment for carbapenem-resistant *K. pneumoniae* with CAZ-AVI. Shields et al. also found all 10 *K. pneumoniae* isolates expressed TEM-1, SHV-11, and OXA-9 β-lactamases [6]. In another study, Venditti et al. collected isolates from six different hospitals in Rome and discovered all the isolates had *bla*_KPC_ genes along with *bla*_CTX-M-15_, *bla*_OXA-9_, *bla*_SHV-182_, and *bla*_TEM-1_ [31].

These studies show the potential for non-carbapenemase β-lactamases to be involved with CAZ-AVI resistance. Porin deficiencies are also related to CAZ-AVI resistance [22].

Its rapid dissemination and molecular evolution suggest that there is a strong selective advantage associated with KPC and that it is experiencing strong positive selection. Its association with other β-lactamases makes it uncertain to what extent KPC is responsible for CAZ-AVI resistance and to what extent CAZ-AVI is a selective pressure driving the evolution of *bla*_KPC_. Here we investigate the evolution of KPC, specifically the role of positive selection in its evolution, and provide evidence about what role CAZ-AVI may be playing in its evolution.

## 2. Results

### 2.1. Alignment and Fisher’s Exact Test

We created an alignment of the 82 unique KPC amino acid sequences contained in the CARD database using CTX-M-1 as an outgroup and reverted the amino acid alignment of KPC to a DNA alignment. We used dN/dS ratios to test for positive selection across the length of the gene using Fisher’s exact test to compute significance as described in the methods section. Based on the analysis, there were no significant *p*-values for the whole gene sequence. Taken as a whole, the *bla*_KPC_ gene does not show the signature of positive selection, which makes sense because large regions of the gene are strictly conserved to maintain the β-lactamase function. However, there are regions such as the Ω-loop where it is still possible that there are individual sites under positive selection that could be identified using phylogenetic reconstruction and analysis of individual variable sites to search for positive selection.

### 2.2. Phylogenetic Reconstruction

**Figure 1:**
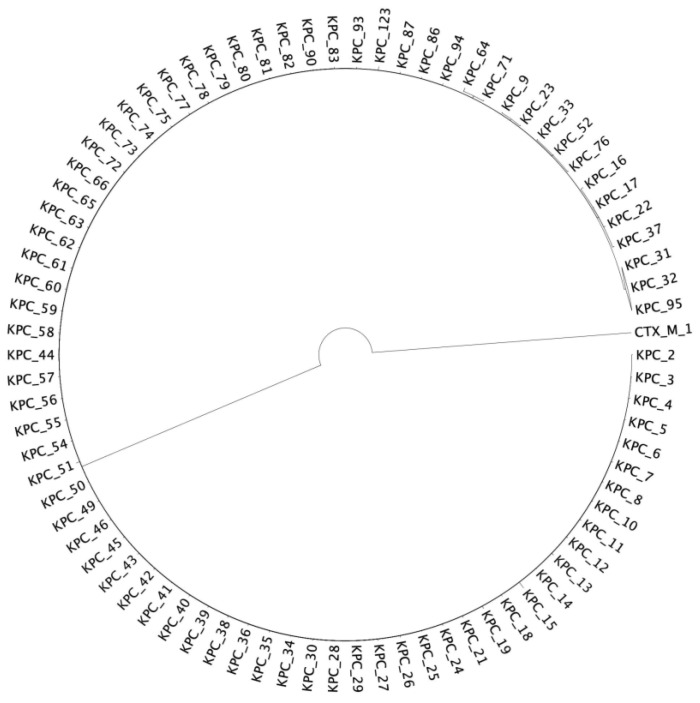
KPC Subtree.

We used MrBayes to reconstruct the *bla*_KPC_ phylogeny (Figure 1) and rooted the tree with the Class A β-lactamase gene *bla*_CTX-M-1_ (AJ310929.1). The consensus KPC phylogeny has short branches, many of which have lengths that are less than one mutation, which reflects fewer differences among KPCs in the phylogeny than in the alignment. Careful inspection of the alignment showed the reason the branch lengths were shorter than one mutation. Although the alignment showed more differences than that, MrBayes does not include indels within its mutation models, and most of the sequence variation in KPC is from indels. MrBayes corrects for multiple mutations at a single site, which is why some branch lengths were shorter than one mutation but still not zero even when the sole source of variation was indels.

Parsimomy handles this type of sequence variation by taking into account all sequence variations, including indels and nucleotide changes. Maximum parsimony reconstruction [32] was used and resulted in two trees with equal parsimony scores (Supplemental data: Figures S1 and S2).

### 2.3. Analysis of Sites under Positive Selection

In order to correctly identify sites and correctly compare them with existing reports, we aligned the KPC amino acid sequence to the Ambler alignment to assign standard homology-based numbering of amino acids to KPC (Supplemental data: Ambler_Protein_1.nexus).

All of our trees vary in topology. To make sure that our results are true to the alignment data and the evolutionary history of *bla*_KPC_, our investigation used all three phylogenies. Using the variable sites in our *bla*_KPC_ alignment, we searched for positive selection at individual sites for each phylogeny. Individual nucleotides show evidence of positive selection when they occur within the phylogeny independently multiple times. We quantified how many times each single nucleotide polymorphism (SNP) occurred in the alignment and then used the phylogenies to quantify how many times each occurred independently [33] across all three trees and found the following mutations have occurred multiple times in all our phylogenetic reconstructions: P104, W105, A120, R164, L169, A172, D179, V240, Y241 (2 out of 3), T243, Y264, and H274. Table 1 shows how many times each SNP occurred independently in each phylogeny. Multiple independent occurrences of mutations indicate numerous occurrences of positive selection.

There are many published reports of the mutations that correspond with CAZ-AVI resistance. The substitutions at sites R164, L169, A172, D179, V240, Y241, T243, Y264, and H274 correspond with CAZ-AVI resistance [13]. Given that all the substitutions identified in this analysis correspond with CAZ-AVI resistance (except for those at sites P104, W105, and A120), it seems likely that CAZ-AVI consumption is a major selective pressure for positive selection in *bla*_KPC_ evolution.

The phylogeny was constructed using MrBayes and Figtree for visualization. The KPC genes 2–123 were rooted with CTXM-1; the figure below is the subtree of KPC only.

## 3. Discussion

We identified the variable sites under positive selection within our three phylogenetic reconstructions of KPC and consistently found evidence of positive selection at amino acids P104, W105, A120, R164, L169, A172, D179, V240, Y241, T243, Y264, and H274. The substitutions P104R, R164P, L169M, A172D, D179Y, V240G, T243A, Y264N, and H274Y affect resistance when they arise as single substitutions in KPC. There is evidence that many of these residues have direct effects on the resistance phenotypes conferred by KPC enzymes [13,34]. Substitutions P104, W105, and A120 contribute to ceftazidime resistance; however, there is no evidence that substitutions at these sites contribute to CAZ-AVI resistance [34].

There are some enzymatic studies of how these substitutions function to affect ceftazidime resistance. Substitutions P104R and V240G affect the hydrolysis of ceftazidime in KPC-4 via the interaction of R104 with the C7 carboxylate of ceftazidime and the positioning of W105 [35]. Residues 104, 105, and 240 delimit the active site. Residue 105 is very important in the recognition of ligands [34], and W105 may favor the carbapenem substrate interactions [13]. Based on a study in 2015 by Mehta et al., P104R and P104L contribute to ceftazidime resistance [34,36]. P104R substitutions lead to increased ceftazidime MICs (>256). Therefore, the substitutions at positions 104, 105, and 120 influence the KPC enzymes and substrates; however, CAZ-AVI resistance is not linked to these substitutions.

There are many other sites under positive selection that are involved with CAZ-AVI resistance. Other studies have found residues R164, L169, A172, D179, V240, Y241, T243, Y264, and H274 to be responsible for CAZ-AVI resistance. Specific substitutions involved in CAZ-AVI resistance are R164S, L169H/P/Q, A172D/T/V, D179A/G/N/V, V240A/G, Y241H/S/N, T243S/M/A/P, Y264H, H274N, and many other insertion/deletion mutations [13]. These substitutions are consistent with our results (Table 1).

Shields et al. identified the most common mutation with CAZ-AVI-resistant *K. pneumoniae* isolates as the D179Y substitution alone or in combination with other substitutions such as T243M [6,37,38]. A study by Zhang et al. reported that mutations in the *bla*_KPC_ gene were in fact a leading cause of resistance after CAZ-AVI treatment. The most common substitution was D179Y due to the diminution in efficiency of avibactam combined with the residual activity of ceftazidime hydrolysis. D179N is reported to increase the binding affinity of ceftazidime, therefore preventing the binding of avibactam. They also found that L169P, V240G, and T243M mutations play a crucial role in CAZ-AVI resistance too [39].

The mechanisms of some of these substitutions have been solved. All the substitutions in positions 164–179 are in the KPC Ω loop, which enables them to improve the affinity of KPC for ceftazidime and prevent the binding of avibactam [13,40]. Parwana et al. found that substitutions R164S and D179N/Y/A/Q in the Ω loop region of KPC-2 confer resistance to CAZ-AVI [41]. Another study by Hemarajata revealed emerging CAZ-AVI resistance in KPC-2 was a result of substitution L169P in the Ω loop [42]. Amino acid D179 is in the Ω loop and contributes to CAZ-AVI resistance by destabilizing the Ω loop, which enhances the activity against ceftazidime [43]. R164 also contributes to CAZ-AVI resistance by destabilizing the salt bridge with D179 [44].

These biochemical studies, combined with our phylogenetic analysis, indicate that *bla*_KPC_ evolution is driven by strong selective pressure from CAZ-AVI, which further confirms the importance of this gene for CAZ-AVI resistance, even if other β-lactamases are involved.

Our phylogenetic analysis of KPC also raises interesting technical challenges associated with the phylogenetic reconstruction of rapidly evolving antibiotic resistance genes. MrBayes is a powerful program for Bayesian inference for phylogenies. MrBayes does not take indels into account; therefore, we reconstructed the evolution of *bla*_KPC_, which is rich with indels, using parsimony to give equal weight to indels and SNPs. While this is a less sophisticated model of evolution, it more completely represents our data. With all three phylogenies, there were identical sets of sites under positive selection.

An additional challenge in reconciling our data with previously published reports was the inconsistent numbering of homologous sites in the KPC protein. The Ambler alignment provides a consistent numbering scheme for class A β-lactamases. There are several published inconsistencies between the Ambler alignment and KPC numbering [8]. Therefore, it is important when using Ambler numbering to freshly align the newly emerged resistance genes, such as KPC, to the complete Ambler alignment to determine the standard homology-based numbering (Supplemental: Ambler_Protein_1.nexus).

## 4. Methods

The Comprehensive Antibiotic Resistance Database (CARD; https://card.mcmaster.ca (accessed on 9 March 2023)) is an armamentarium of molecular and sequence data for antibiotic resistance [45]. The KPC gene family is composed of 82 genes with unique sequences in the CARD database. There are many more KPCs; however, not all sequences have been published. We downloaded the *bla*_KPC_ gene family on 9 March 2023, in a FASTA file from CARD. A total of 81 KPC genes were downloaded. The FASTA file was then uploaded to the Molecular Evolutionary Genetics Analysis (MEGA) software version 11. MEGA software was used for comparative sequence analysis [46].

### 4.1. Alignment

We used multiple sequence comparison by log-expectation (MUSCLE) to align the KPC protein sequences on MEGA [47] using the following default parameters: gap opening enalty −400.00, gap extension penalty 0.00, and 16 maximum iterations. The cluster method iterations used the unweighted pair-group method with arithmetic mean (UPGMA). After protein alignment, we reverted the aligned protein sequences back to nucleotide sequences for further analysis.

### 4.2. Test for Selection

Fisher’s exact test was used to test for a significant difference between the numbers of nonsynonymous and synonymous mutations. When Fisher’s exact test is used for selection, the null hypothesis is that Dn = Ds, and the significant *p*-value would reject that hypothesis. The total number of synonymous and nonsynonymous substitutions was too small, and the large sample size Z-tests were too liberal in rejecting the null hypothesis [32]. The *p*-values are not to be trusted for large sample-based statistics when your sample size is small [48]. The only methods that can be used are Nei-Gojobori and Modified Nei-Gojobori because they require the direct computation of the numbers of synonymous and nonsynonymous differences, along with the number of synonymous and nonsynonymous sites [32]. Using an exact distribution leads to *p*-values that can be conservative [48]. Nonetheless, the analysis had no significant *p*-values for the whole gene sequence. This led us to use phylogenetic reconstruction with the analysis of individual variable sites to investigate positive selection.

### 4.3. β-Lactamase Numbering Scheme

Previously published KPC alignments claim that the Ambler numbering was inconsistent with the original Ambler alignment [8]. To apply the homology-based numbering scheme developed by Richard Penry Ambler to the KPC protein sequences, we downloaded and aligned 17 of the sequences used in the original Ambler alignment [4]. All sequences were downloaded from GenBank (Table 2). KPC was added after recreating the Ambler alignment and confirming it matched the published alignment (Supplemental data: Ambler_Protein_1.nexus). This enabled us to determine and apply the standard numbering to KPC. The specifics of aligning KPC to the Ambler alignment are that we added single amino acid (1 codon) gaps at positions 58 and 253. This caused the KPC sequence to align exactly with the Ambler alignment.

### 4.4. Phylogenies

Our *bla*_KPC_ alignment contained a few informative sites, and they comprised both point mutations and indels. Therefore, it was necessary to use multiple phylogenetic methods to identify which sites are under positive selection. First, we reconstructed the phylogeny with the MrBayes program, using the default parameters, and ran the analysis for 3.2 million generations until the standard deviation was 0.009243. Figtree software visualized the consensus tree (Figure S2). We rooted the *bla*_KPC_ tree with *bla*CTX-M-1 (AJ310929.1) as the outgroup. We generated the maximum parsimony trees using the default settings in MEGA11; two trees were constructed. We then constructed 500 bootstrap replicates using the MEGA default parameters.

## Figures and Tables

**Table 1:** Amino Acid Substitutions in KPC Enzyme that Occur Independently Multiple Times.

	Position	6	8	13	18	34	49	62	89	92	93	104	105	120	147	163	164	165	169	170	171	172	179	180	191	202	207	240	241	243	254	264	270	274	292	293	294
	Wildtype	R	V	L	A	A	M	A	G	D	T	P	W	A	G	D	R	W	L	N	S	A	D	T	Q	P	F	V	Y	T	T	Y	K	H	V	N	G
KPC-2	AY034847.1																																				
KPC-3	AF395881.1																																	Y			
KPC-4	EU447304.1											R																G									
KPC-5	EU400222.2											R																									
KPC-6	EU555534.1																											G									
KPC-7	EU729727.1						I																											Y			
KPC-8	FJ234412.1																											G						Y			
KPC-9	FJ624872.1																											A						Y			
KPC-10	GQ140348.1											R																						Y			
KPC-11	HM066995.1											L																									
KPC-12	HQ641421.1																		M																		
KPC-13	HQ342889.1									G																								Y			
KPC-14	JX524191.1																																				
KPC-15	KC433553.1											R		L	K													G						Y			
KPC-16	KC465199.1																									S	L										
KPC-17	KC465200.1																										L										
KPC-18	KP681699.1		I																																		
KPC-19	KJ775801.1																																	Y		T	
KPC-21	NG_049254.1												R																								
KPC-22	KM379100.1												G														L										
KPC-23	MH450213.1																											A						Y			
KPC-24	KR052099.1	P																																			
KPC-25	NG_051167.1																																				
KPC-26	KX619622.1				S																																
KPC-27	KX828722.1												R																					Y			
KPC-28	KY282958.1																																	Y			
KPC-29	KY563764.1																																	Y			
KPC-30	KY646302.1	H																																			
KPC-31	MAPH01000113.1																						Y											Y			
KPC-32	MAPO01000050.1																						Y							M				Y			
KPC-33	CP025144.1																						Y														
KPC-34	KU985429.1																																				
KPC-35	MH404098.1																		P																		
KPC-36	MH593787.1															E																		Y			
KPC-37	MH718730.1																	R									L										
KPC-38	MK098861.1																																	Y	A		
KPC-39	MK118771.1																					T												Y			
KPC-40	QRBR01000058.1																													S				Y			
KPC-41	MK497255.1			?																														Y			
KPC-42	MK467612.1																														A						
KPC-43	MK628511.1																								R												
KPC-44	NG_065427.1																																				
KPC-45	MN104596.1										K																										
KPC-46	MN267701.1																		P															Y			
KPC-49	MN619655.1																S																	Y			
KPC-50	MN654342.1																																	Y			
KPC-51	MN725731.1																						N						H					N			
KPC-52	MN725732.1																						Y														
KPC-54	MN854706.1							S																													
KPC-55	MT028409.1																															N					
KPC-56	MT040751.1																																	Y			W
KPC-57	MT358626.1																						V														
KPC-58	NG_070177.1																																				
KPC-59	NG_070178.1								D																												
KPC-60	NG_070179.1													T																							
KPC-61	NG_070180.1																				P													Y			
KPC-62	NG_073465.1																		Q															Y			
KPC-63	NG_073466.1																												S					Y			
KPC-64	NG_073467.1																							S						A		H		Y			
KPC-65	NG_073468.1																																	Y			
KPC-66	NG_070739.1																																	Y			
KPC-71	NG_070895.1																							S													
KPC-72	NG_070740.1																					D															
KPC-73	NG_070741.1																																				
KPC-74	NG_070742.1																																				
KPC-75	NG_070743.1			F																																	
KPC-76	NG_070896.1																						Y														
KPC-77	NG_070897.1																P																				
KPC-78	NG_071204.1																						A														
KPC-79	NG_071205.1																																				
KPC-80	NG_073469.1																																				
KPC-81	NG_073470.1																																				
KPC-82	NG_073471.1																																				
KPC-83	MW581775.1					T																															
KPC-86	MZ067229.1																						G														
KPC-87	MZ067230.1																													A							
KPC-90	MZ570431.1																																				
KPC-93	MZ569034.1																																				
KPC-94	MZ646140.1																			H														Y			
KPC-95	MZ646141.1																					T	Y											Y			
KPC-123	ON012820.1																																A				
	MrBayes											5	3	2			2		4			3	6					5	1	4		2		27			
	Parisomony1											3	3	2			2		4			3	7					4	2	4		2		10			
	Parisomony2											4	3	2			2		4			3	4					4	2	4		2		12			

**Table 2:** Seventeen of the sequences used in the original Ambler alignment and corresponding accession numbers. We also include the KPC-2 sequence used for the alignment and Ambler numbering.

Species/Gene	Accession	Citation
*Klebsiella pneumoniae*	NG049268.1	[49]
PIT-2 (SHV-1)	P0AD63.1	[50]
R-TEM	J01749.1	[51]
PSE-4	J05162	[52]
*Rhodopseudomas capulate* (*ampR* gene)	X1579.1	[53]
*Actinomadura*	NG_047541.1	[54]
*Bacillus cereus 569H*	NG_047482.1	[55]
*Bacillus cereus 5/B*	M12607.1	[56]
*Bacillus cereus III*	M15195	[57]
*Bacillus licheniformis penicillinase*	V00093.1	[58]
*Streptomyces badius*	M34178.1	[58]
*Streptomyces cacaoi Ulg*	BAA14224.1	[59]
*Klebsiella oxytoca*	M27459.1	[60]
*Staphylococcus aureus*	X04121	[61]
*Streptomyces albus*	NG047481.1	[62]
*Streptomyces lavendulae*	M34180.1	[58]
*Streptomyces fradiae*	M34179.1	[58]
KPC-2	NG_049253	[63]

## Data Availability

The data supporting this research are publicly available through Genbank. Accession numbers are given.

## Supplementary Materials

The following supporting information can be downloaded at: https://www.mdpi.com/article/10.3390/antibiotics13010040/s1, Figure S1: Parsimony reconstruction of KPC phylogeny; Figure S2: Parsimony reconstruction of KPC phylogeny; Supplemental Data: Ambler_Protein_1.nexus: Ambler alignment including KPC.
