# Supplementary figures and images for: The Klebsiella pneumoniae carbapenemase (KPC) β-Lactamase Has Evolved in Response to Ceftazidime Avibactam
Source: Antibiotics (Basel). 2023 Dec 31;13(1):40. doi: 10.3390/antibiotics13010040 (PMC10812414; doi:10.3390/antibiotics13010040)

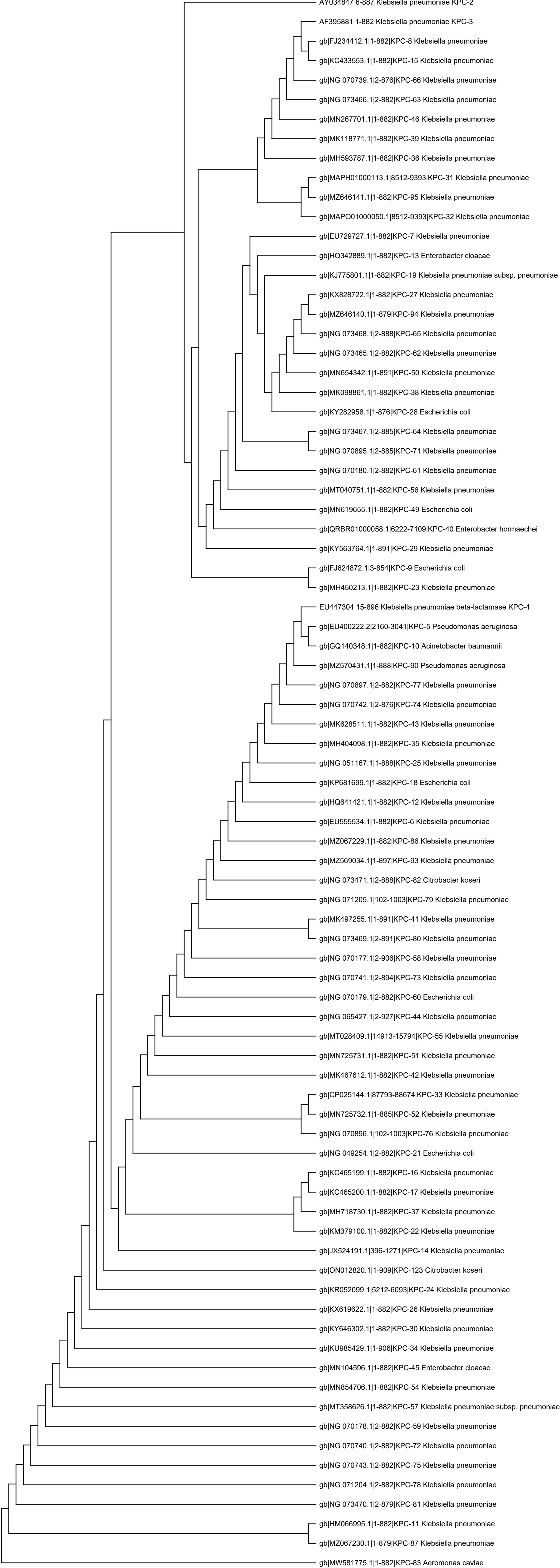

Supplement: Supplementary file 1 [file antibiotics-13-00040-s001.zip › Pars 1.pdf]

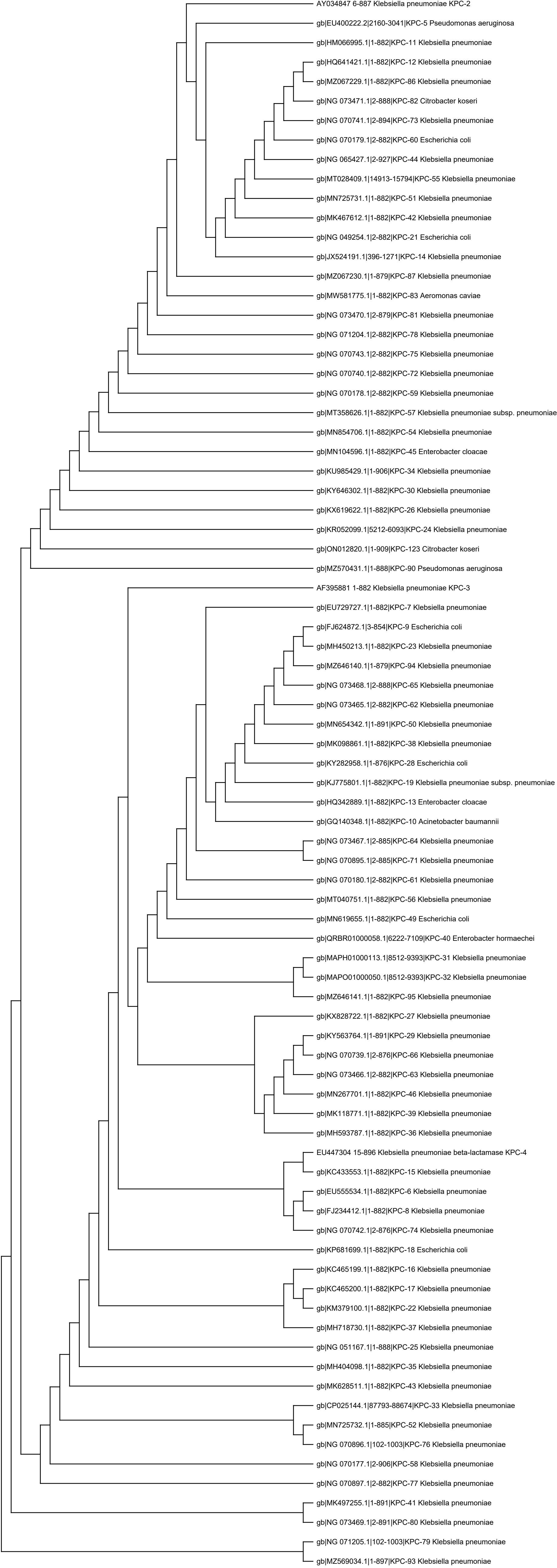

Supplement: Supplementary file 1 [file antibiotics-13-00040-s001.zip › Pars 2.pdf]
